# Correction: Protein profile analysis of tear fluid with hyphenated HPLC-UV LED-induced fluorescence detection for the diagnosis of dry eye syndrome

**DOI:** 10.1039/d3ra90084c

**Published:** 2023-09-13

**Authors:** Sphurti S. Adigal, Sulatha V. Bhandary, Nagaraj Hegde, V. R. Nidheesh, Reena V. John, Alisha Rizvi, Sajan D. George, V. B. Kartha, Santhosh Chidangil

**Affiliations:** a Centre of Excellence for Biophotonics, Department of Atomic and Molecular Physics, Manipal Academy of Higher Education Manipal Karnataka India 576104 santhosh.cls@manipal.edu; b Department of Ophthalmology, Kasturba Medical College, Manipal, Manipal Academy of Higher Education Manipal Karnataka India-567104; c Ato-gear BV Schimmelt 28 5611 ZX Eindhoven Netherlands; d Centre for Applied Nanoscience, Department of Atomic and Molecular Physics, Manipal Academy of Higher Education Manipal Karnataka India 567104

## Abstract

Correction for ‘Protein profile analysis of tear fluid with hyphenated HPLC-UV LED-induced fluorescence detection for the diagnosis of dry eye syndrome’ by Sphurti S. Adigal *et al.*, *RSC Adv.*, 2023, **13**, 22559–22568. https://doi.org/10.1039/D3RA04389D.

The authors regret that the incorrect caption for **Fig. 2** was included in the original article. The correct version of **Fig. 2** is presented here.


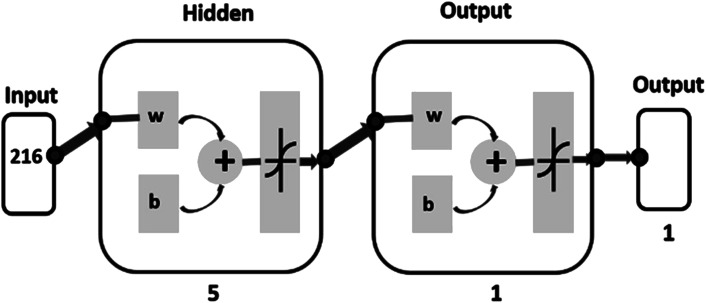
 **Fig. 2** Network architecture.

The authors also regret that affiliations *b* and *d* were incorrectly shown in the original manuscript. The corrected list of affiliations is as shown here.

The Royal Society of Chemistry apologises for these errors and any consequent inconvenience to authors and readers.

## Supplementary Material

